# Loneliness and Distress in the Aftermath of the COVID-19 Pandemic: A Cross-Sectional Study of German University Students

**DOI:** 10.32872/cpe.14365

**Published:** 2025-05-28

**Authors:** Joanna J. Hunsmann, Florian Weck, Julia Wendt, Franziska Kühne

**Affiliations:** 1 Department of Clinical Psychology and Psychotherapy, University of Potsdam, Potsdam, Germany; 2 Department of Emotion- and Biopsychology, University of Potsdam, Potsdam, Germany; Friedrich-Alexander-Universität Erlangen-Nürnberg, Erlangen, Germany

**Keywords:** mental health, psychological distress, loneliness, emotion regulation, health behaviors, COVID-19 pandemic, university students

## Abstract

**Background:**

Characterized by uncertainty and recurring periods of social isolation, the COVID-19 pandemic resulted in increases of loneliness and distress in young adults, such as university students. Despite the lifting of the last restrictions in Germany in April 2023, the state of mental health in vulnerable groups after the three-year global crisis remains to be investigated. Therefore, we aimed to assess university students’ mental health after the pandemic.

**Method:**

Between April and July 2023, *N* = 886 university students throughout Germany participated in a fully anonymous cross-sectional online survey. Psychological distress (BSI; Brief Symptom Inventory), loneliness (LS-SOEP; Loneliness Scale), and emotion regulation strategies (ERQ; Emotion Regulation Questionnaire) were assessed by standardized questionnaires, and mental health was compared to a survey of students in April 2020 (*N* = 1,062).

**Results:**

Unexpectedly, we found higher levels of distress in 2023 than in 2020. Overall, *R^2^*_adj_ = 41% of variance in psychological distress was accounted for in a multiple linear regression, with loneliness emerging as the most important predictor. Additionally, emotion regulation, gender identity, and health behaviors such as keeping daily routines, sufficient sleep, and regular exercise were significant predictors. Analyses of variance (ANOVAs) revealed that students with past or present mental health conditions were significantly lonelier than those without.

**Conclusion:**

These findings highlight the ongoing mental health challenges of university students in the aftermath of the COVID-19 pandemic, identifying non-binary and female students, as well as students with current or past mental health conditions as particularly lonely and distressed.

## Highlights

- This study is among the first to investigate post-pandemic mental health in German university students.
- Differences in loneliness were found by gender, with non-binary students particularly affected.
- Students with current or past mental health conditions reported more loneliness and more distress.
- Health behaviors and adaptive emotion regulation predicted better mental health.

Impacting various domains of life, the COVID-19 pandemic has been widely recognized not only as a threat to physical, but also to mental health (Bower et al., 2023; Gruber et al., 2021; World Health Organization, 2020). Across borders, it was accompanied by increases in psychological distress and symptoms of depression and anxiety (Benke et al., 2020; Bower et al., 2023). Characterized by periods of social isolation, the COVID-19 pandemic has also seen a notable rise in loneliness (Ernst et al., 2022). University students, often confronted with financial instability, academic pressures, and instability in social networks, are considered particularly vulnerable to mental health deterioration (Auerbach et al., 2018; Ochnik et al., 2021).

In Germany, university students’ mental health was affected by frequently changing study conditions (Matos Fialho et al., 2021). Typically associated with older age, a rise in loneliness in young adults in Germany during the COVID-19 pandemic has been highlighted (Lepinteur et al., 2022; Werner et al., 2021). This is alarming since social contacts are considered especially important in this life phase. Defined as subjective experience of distress resulting from perceived deficiencies in the quantity and quality of social connections (Hawkley & Cacioppo, 2010), loneliness has been tied to a variety of adverse health outcomes (Cacioppo et al., 2010; Hawkley et al., 2009). Early longitudinal studies reported responses of recovery after the lifting of lockdown restrictions, however, levels of loneliness remained notably high (Ahrens et al., 2021; Chandola et al., 2022; Entringer et al., 2020). In Germany, pandemic restrictions (e.g., mask-wearing) were maintained comparatively long, with final regulations ending in early 2023 (Bundesregierung, 2023; see also Appendix A in the Supplementary Materials).

Mauz and colleagues (2023) provide evidence for the deterioration of mental health during later waves of the pandemic, highlighting the negative effect of the war in Ukraine. Given the pandemic duration, added stressors, and the serious threat for mental health given prolonged loneliness, monitoring university students’ mental health in the aftermath of the COVID-19 pandemic remains relevant.

During the pandemic, identifying as female (Benke et al., 2020; Lepinteur et al., 2022), younger age (Benke et al., 2020; Bower et al., 2023), and prior mental health conditions (Benke et al., 2020; Bower et al., 2023; Shevlin et al., 2023) were consistently associated with greater distress. Individuals belonging to a risk group for COVID-19 or experiencing lasting consequences (e.g., post-COVID) were more distressed (Houben-Wilke et al., 2022). Meanwhile, in line with recommendations by health organizations (Inter-Agency Standing Committee, 2020), better mental health was associated with behaviors such as keeping daily rhythms, regular physical exercise, healthy nutrition, and sufficient sleep (Mata et al., 2021; Shanahan et al., 2022; Voltmer et al., 2021). Furthermore, adaptive emotion regulation was associated with less distress and loneliness (Ahrens et al., 2021; Preece et al., 2021). Policymakers and university staff would benefit from understanding which students may be particularly burdened after COVID-19.

In this study, we examined German university students’ mental health in the aftermath of the COVID-19 pandemic. Specifically, we hypothesized that students would report lower levels of psychological distress in spring 2023, after restrictions ended, compared to the lockdown period in 2020. We expected correlations between psychological distress and loneliness, as well as emotion regulation strategies. We hypothesized that loneliness and emotion regulation strategies would be significant predictors for psychological distress, beyond the impact of person-related factors and health behaviors. Finally, we examined the role of students’ prior mental health conditions and hypothesized that students with current or past mental health conditions would report more loneliness, demonstrate less adaptive emotion regulation, and engage less in health-related behaviors compared to others.

## Method

### Procedure

A total of *N* = 886 university students in Germany participated in the cross-sectional online study between April and July 2023. The survey was conducted using the survey tool of the University of Potsdam. Participants were recruited nationally via email through universities’ student councils, with requests to spread the survey link among their students, as well as locally at the University of Potsdam via the participant pool of the Cognitive Sciences and by distribution of flyers at key locations on campus during mental health awareness days.

Inclusion criteria were student status, studying in Germany, age 18 and above, and giving informed consent. The latter was obtained from all participants, ensuring thorough understanding of their rights, the study's objectives, risks, and benefits. Abiding by institutional guidelines of the University of Potsdam, data collection maintained complete anonymity, strictly limiting sociodemographic information asked, and adhering rigorously to national and EU data protection laws. Participant characteristics can be found in Table 1.

### Measures

The questionnaire commenced with a brief assessment of sociodemographic variables, namely gender (female, male, non-binary), age (in categories to further ensure anonymity, e.g., 21-25), relationship status, and study term.

#### COVID-19-Related Variables

Participants were asked how often they had been infected with the COVID-19 virus and whether they belonged to one of the following three groups, later referred to as COVID-related group status: at-risk for severe infection, having experienced a severe infection, and experiencing lasting effects of a past infection. Participants were also asked about the extent to which they felt burdened by the COVID-19 pandemic, the war in Ukraine, the energy crisis, and the climate crisis. Answers were given on a 5-point Likert scale, ranging from 1 (*not at all*) to 5 (*very strongly*).

#### Psychological Distress

Psychological distress was assessed with the German version of the Brief Symptom Inventory (BSI; Derogatis & Melisaratos, 1983; Franke, 2000). Consisting of 53 items, it measures distress on nine dimensions. General psychological distress is indicated by a global score, the General Severity Index (GSI). The instrument uses a 5-point Likert scale, ranging from 0 (*not at all*) to 4 (*extremely*). Higher levels reflect greater distress. To calculate the GSI, the sum of all item scores is divided by 53, the number of items. In the present study, Cronbach’s α was .96.

#### Loneliness

The German version of the 3-item Loneliness Scale (LS-SOEP; Hawkley et al., 2015; Hughes et al., 2004) was used to assess loneliness, which is derived from the UCLA Loneliness Scale (Russell, 1996). Responses to the items were given on a 5-point Likert scale, ranging from 0 (*never*) to 4 (*very often*). We used the mean value of given responses (Luhmann & Hawkley, 2016), with higher values indicating greater loneliness. In this sample, Cronbach’s α was .77.

#### Emotion Regulation

Emotion regulation was measured with the German version (Abler & Kessler, 2009) of the Emotion Regulation Questionnaire (ERQ; Gross & John, 2003). It contains 10 items that measure how a person deals with positive and negative emotions. The scale *Suppression* consists of six items and the scale *Reappraisal* consists of four items rated from 1 (*strongly disagree*) to 7 (*strongly agree*). In our sample, Cronbach’s α was .81 for *Reappraisal* and .75 for *Suppression*.

#### Health-Related Behaviors

Six items regarding health behaviors were self-developed by adapting recommendations of the of the Inter-Agency Standing Committee of the United Nations (2020). In line with previous studies during the COVID-19 pandemic, self-reported regularity of exercise, daily living rhythms, sufficient sleep, healthy dietary habits, mindfulness practices, and alcohol consumption were recorded (Chen et al., 2020; Koob et al., 2021; Mata et al., 2021; Petzold et al., 2020). Answers were given on a 4-point scale, ranging from 1 (*disagree*) to 4 (*agree*), with higher values indicating stronger practice of health behaviors (see Appendix C, Supplementary Materials, for full item list).

#### Mental Health Conditions

Students were queried regarding prior mental health conditions (1 = *Yes, I am currently undergoing treatment for it*; 2 = *Yes, I received treatment for it in the past*; 3 = *No*). If affirmed, participants were asked to specify whether there had been a worsening of symptoms since the pandemic.

### Comparison Data

To compare post-pandemic levels of psychological distress to students’ mental health in the beginning of the COVID-19 pandemic, we used data from a survey conducted at our university in April 2020 (Löscher, 2020). A total of *N* = 1,062 (*n* = 706, 66.5% female; *n* = 345, 32.5% male; *n* = 11, 1% non-binary) university students in Germany participated in this cross-sectional online survey. Participants were aged 18 to 51 years (*M* = 24.01; *SD* = 4.68). The procedure was analog to the assessment detailed for 2023, except for the distribution of flyers on campus.

### Statistical Analysis

All analyses were conducted in R (R Core Team, 2023). There were no missing data. However, for the study term question, some exclusions were made due to non-digit entries (e.g., “bachelor”, 23 cases). The level of significance for all analyses was α = .05. Analyses were conducted as previously registered, with minor deviations detailed below due to unmet assumptions (Hunsmann & Kühne, 2023S, March 31). First, we computed descriptive statistics. In exploratory manner, we examined the correlations between perceived burdens and psychological distress, applying the Bonferroni correction for multiple comparisons (Armstrong, 2014). We also explored differences in loneliness by gender.

Proceeding with our hypotheses, we examined the relationships between psychological distress, loneliness, and emotion regulation strategies, using Spearman’s rank correlations due to violations of the assumption of normality. To better understand how distressed students were in the aftermath of the COVID-19 pandemic, we used a Mann-Whitney *U*-test to compare students’ levels of psychological distress in both samples. The Mann-Whitney *U*-test was conducted due to violations in the assumptions of equal variances and normality. Effect sizes are reported, with *r* = .1 considered a small, *r* = .3 a moderate, and *r* = .5 a large effect (Cohen, 1988).

Next, we conducted hierarchical multiple regression analyses to predict psychological distress from loneliness and emotion regulation strategies, while controlling for health behaviors and person-related factors. First, we considered a baseline model with health behaviors and person-related factors. In a second step, we added the emotion regulation strategies suppression and reappraisal as predictors. Similarly, we added the predictor loneliness to the baseline model. Finally, we computed a multiple regression model predicting psychological distress from all variables. As the assumption of homoskedasticity was not confirmed for all regression models, we employed heteroskedasticity-consistent, robust estimations using the HC3 method in all regression analyses (Hayes & Cai, 2007; Long & Ervin, 2000; White, 1980). Where the assumption of normality of residuals was violated, we conducted bootstrapped regression analyses with 5000 bootstrap samples to assess the stability of the original regression models’ results. If not specified otherwise, the bootstrapped regression analyses supported the initial models.

Finally, we conducted analyses of variance (ANOVAs) to investigate differences in loneliness, emotion regulation strategies, and health behaviors between participants depending on their history of mental health conditions. Post hoc contrasts were computed using the Tukey Honestly Significant Difference (HSD) test. Effect sizes were calculated using omega squared (ω^2^) as recommended by Kroes and Finley (2023). Field’s (2013) recommendation for interpreting ω^2^ was used (ω^2^ ≥ 0.01 small, ω^2^ ≥ 0.06 medium, ω^2^ ≥ 0.14 large).

## Results

### Sample

An overview over sociodemographic information is provided in Table 1, experiences related to the COVID-19 pandemic in Table 2.

**Table 1:** Sample Characteristics

Characteristic	overall *N* = 886 *n* (%)	female *N* = 551 *n* (%)	male *N* = 307 *n* (%)	non-binary *N* = 28 *n* (%)
Age				
18-20	165 (18.6%)	108 (19.6%)	51 (16.6%)	6 (21.4%)
21-25	498 (56.2%)	311 (56.4%)	171 (55.7%)	16 (57.1%)
26-30	155 (17.5%)	94 (17.1%)	57 (18.6%)	4 (14.3%)
31-35	32 (3.6%)	17 (3.1%)	15 (4.9%)	0 (0.0%)
36-40	22 (2.5%)	11 (2.0%)	10 (3.3%)	1 (3.6%)
41-45	6 (0.7%)	5 (0.9%)	1 (0.3%)	0 (0.0%)
46-50	3 (0.3%)	1 (0.2%)	2 (0.7%)	0 (0.0%)
51+	5 (0.6%)	4 (0.7%)	0 (0.0%)	1 (3.6%)
Education (in semesters)				
*M* (*SD*)	6.13 (3.56)	6.09 (3.43)	6.25 (3.78)	5.59 (3.40)
Range	1.00, 24.00	1.00, 18.00	1.00, 24.00	1.00, 14.00
Marital status				
Divorced	1 (0.1%)	1 (0.2%)	0 (0.0%)	0 (0.0%)
Married	32 (3.6%)	27 (4.9%)	4 (1.3%)	1 (3.6%)
Relationship	355 (40.1%)	243 (44.1%)	103 (33.6%)	9 (32.1%)
Single	497 (56.1%)	280 (50.8%)	200 (65.1%)	17 (60.7%)
Widowed	1 (0.1%)	0 (0.0%)	0 (0.0%)	1 (3.6%)
Mental health condition				
No	634 (71.6%)	383 (69.5%)	241 (78.5%)	10 (35.7%)
Current	123 (13.9%)	88 (16.0%)	27 (8.8%)	8 (28.6%)
Previous	129 (14.6%)	80 (14.5%)	39 (12.7%)	10 (35.7%)

**Table 2:** COVID-19-Related Variables

Characteristic	*N* = 886, *n* (%)
Number of infections with COVID-19	
0	165 (18.6%)
1	450 (50.8%)
2	227 (25.6%)
3	40 (4.5%)
4	4 (0.5%)
**At-risk group**	75 (8.5%)
**Severe course**	65 (7.3%)
**Lasting consequences (e.g., post-COVID)**	84 (9.5%)

Of those students who had a history of mental health conditions (*n* = 252; 28.4%), either currently or previously received psychotherapy, 58.3% (*n* = 147) reported worsening symptoms since the COVID-19 pandemic. Furthermore, 44.5% (*n* = 394) of participants were classifiable as psychologically distressed.

**Figure 1:**
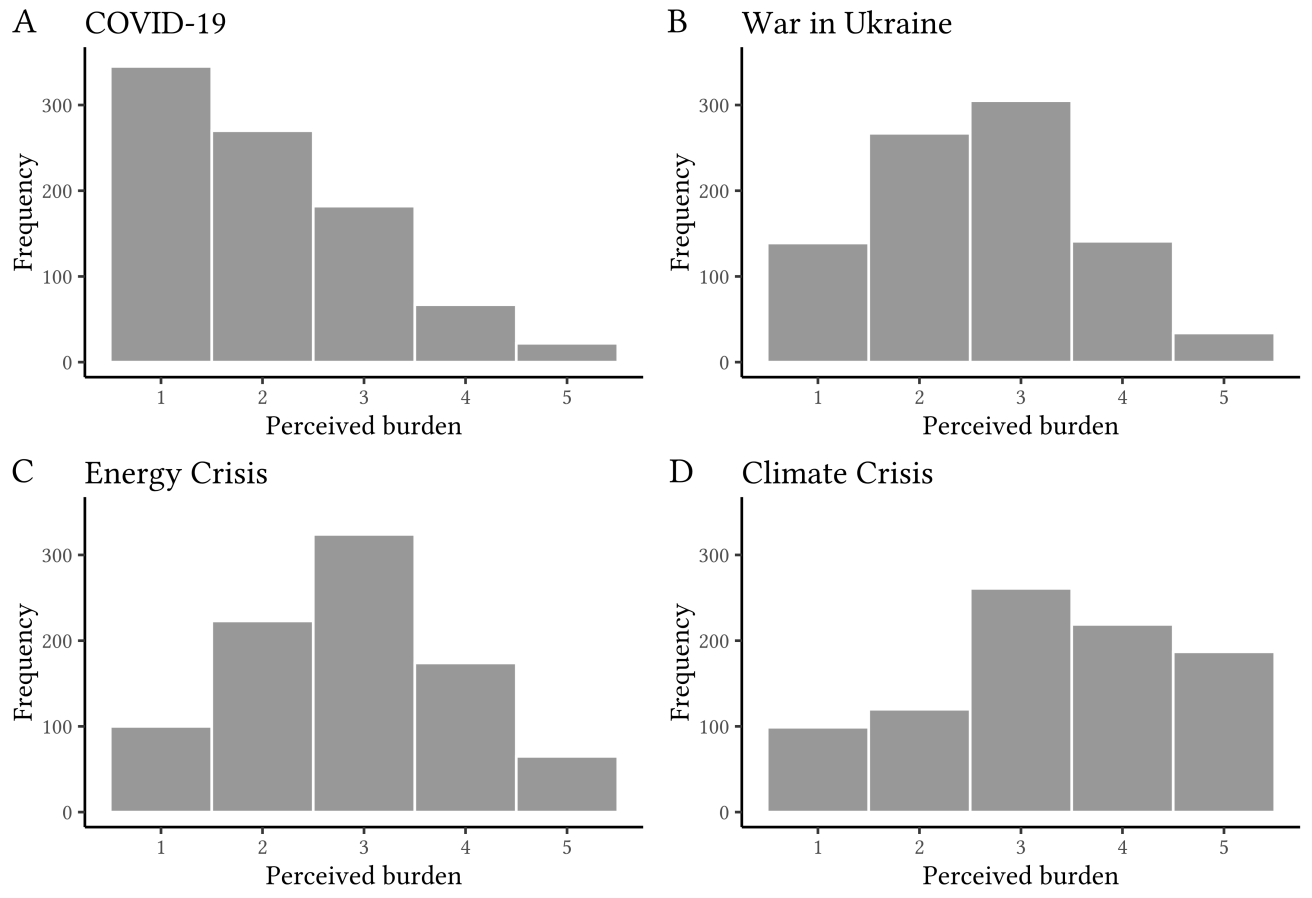
Perceived Burdens Due to Current Events *Note.* Extent to which participants indicated to feel burdened: 1 = *not at all*, 2 = *slightly*, 3 = *somewhat*, 4 = *strongly*, 5 = *very strongly*.

As can be seen in *Figure 1*, 10.1% of students reported feeling *strongly* or *very strongly* burdened by the COVID-19 pandemic. In comparison, the percentage of students reporting to feel *strongly* or *very strongly* burdened by the other stressors were 17.6% for the war in Ukraine, 26.9% for the energy crisis, and 45.8% for the climate crisis. Yet, there was a small but significant correlation between students’ distress and how burdened they felt by the COVID-19 pandemic (*r* = .26, *p* < .001). Likewise, there were small correlations between psychological distress and perceived burdens due to the energy crisis (*r* = .28, *p* < .001), the war in Ukraine (*r* = .25, *p* < .001), and the climate crisis (*r* = .22, *p* < .001, Bonferroni correction *p* < .0125).

Descriptive statistics for psychological distress, loneliness and emotion regulation are provided in Table 3.

**Table 3:** Descriptive Statistics

Characteristic	overall *N* = 886	Mental health conditions		
		no *n* = 634	current *n* = 123	previous *n* = 129
Psychological distress (GSI)				
*M* (*SD*)	0.88 (0.62)	0.79 (0.57)	1.17 (0.67)	1.05 (0.68)
Range	0.00, 3.58	0.00, 3.23	0.08, 3.58	0.04, 3.00
Loneliness (LS-SOEP)				
*M* (*SD*)	1.89 (0.92)	1.77 (0.88)	2.28 (0.95)	2.09 (0.93)
Range	0.00, 4.00	0.00, 4.00	0.00, 4.00	0.00, 4.00
Reappraisal (ERQ)				
*M* (*SD*)	4.30 (1.08)	4.32 (1.06)	4.21 (1.15)	4.33 (1.08)
Range	1.00, 7.00	1.00, 7.00	1.50, 6.50	1.00, 7.00
Suppression (ERQ)				
*M* (*SD*)	3.72 (1.28)	3.73 (1.27)	3.85 (1.30)	3.55 (1.31)
Range	1.00, 7.00	1.00, 7.00	1.00, 6.75	1.00, 6.50

*Note.* GSI = General severity index; LS-SOEP = 3-item Loneliness Scale; ERQ = Emotion Regulation Questionnaire.

A one-way ANOVA revealed a small yet significant difference in loneliness by gender, *F*(2, 883) = 8.37, *p* < .001, ω^2^ = .016, 95% CI [.004, .039]. Post hoc contrasts showed that participants identifying as non-binary were significantly lonelier (*M* = 3.5, *SD* = 0.98) than participants identifying as female (*M* = 2.92, *SD* = 0.88) or male (*M* = 2.79, *SD* = 0.96). As for differences in loneliness by COVID-19 related group status, a *t*-test revealed that participants at risk for a severe course of infection reported significantly higher loneliness (*M* = 3.21, *SD* = 0.92) than participants not belonging to a risk-group (*M* = 2.86, *SD* = 0.91; *t*(884) = -3.19, *p* = .001, *d* = 0.39), and that participants who reported lasting consequences of a past infection reported significantly higher loneliness (*M* = 3.09, *SD* = 0.95) than participants without post-COVID ramifications (*M* = 2.87, *SD* = 0.91), *t*(884) = -2.10, *p* = .036, *d* = 0.24.

### Difference in Psychological Distress

The Mann-Whitney *U*-test revealed a significant difference in psychological distress between students in 2020 and 2023, *U* = 332516, *z* = -8.211, *p* < .001, *r* = .19, with students in 2023 reporting significantly higher distress (*Mdn* = 0.74) than students in 2020 (*Mdn* = 0.45).

### Psychological Distress in Relation to Loneliness and Emotion Regulation

There was a large positive correlation between psychological distress and loneliness, *r*_s_(884) = .56, *p* < .001. A small negative correlation was found between psychological distress and reappraisal, *r*_s_(884) = -.18, *p* < .001, whereas distress and suppression were positively correlated, *r*_s_(884) = .23, *p* < .001.

The baseline multiple linear regression model predicting psychological distress from health behaviors and person-related factors was statistically significant, explaining 17% of variance, see Table D1 (Appendix D, Supplementary Materials). Adding the emotion regulation strategies to the baseline model resulted in a statistically significant model explaining 24% of variance in psychological distress, see Table D2 (Appendix D). The model was better than the baseline model, Δ*R*^2^ = .066, *F*(2, 866) = 38.5, *p* < .001. The strategy of suppression predicted higher psychological distress, and the strategy of reappraisal lower distress. The bootstrapped regression analysis closely aligned with initial model, however, belonging to the age group 36-40 was corrected as a nonsignificant predictor.

Similarly, the predictor loneliness was added to the baseline model, resulting in a statistically significant model accounting for 39% of the variance in psychological distress, see Table D3 (Appendix D). Here, an increase in loneliness was associated with an increase in psychological distress. The model with loneliness was significantly better than the baseline model, Δ*R*^2^ = .209, *F*(1, 867) = 301.87, *p* < .001.

Lastly, an exploratory regression model with all predictors accounted for *R*^2^ = 41% of variance in psychological distress, see Table 4 (see also Table D4, Appendix D).

**Table 4:** Multiple Regression Analysis: Predictors of Psychological Distress (N = 886)

Variable	Standardized β	*SE*	*t*	*p*
(Intercept)		0.17	3.454	< .001
**Loneliness**	.44	0.02	13.225	< .001
**Suppression**	-.10	0.02	-3.407	< .001
**Reappraisal**	.13	0.01	4.659	< .001
Health behaviors				
Daily structure	-.07	0.02	-2.244	0.025
Healthy nutrition	.02	0.03	0.757	0.449
Regular exercise	-.06	0.02	-2.086	0.037
Mindfulness practice	.05	0.02	1.630	0.104
Sufficient sleep	-.16	0.02	-5.128	< .001
Limiting alcohol	-.03	0.02	-1.241	0.215
Age^a^				
21-25	-0.02	0.04	-0.708	0.479
26-30	0.02	0.06	0.456	0.648
31-35	0.00	0.10	0.151	0.880
36-40	-0.04	0.11	-1.420	0.156
41-45	0.00	0.13	-0.196	0.845
46-50	-0.02	0.18	-1.429	0.153
51+	0.01	0.48	0.226	0.821
Gender^b^				
Male	-.13	0.04	-4.564	< .001
Non-binary	.02	0.15	0.508	0.612
Therapy^c^				
Current	.08	0.06	2.654	0.008
Previous	.09	0.05	2.960	0.003
*R*^2^ ($R_{\mathrm{adj}}^{2}$)	.442 (.409)			
*F*	31.630			
*p*	< .001			

^a^reference category: 18-20. ^b^reference category: female. ^c^reference category: no therapy.

While being the strongest of the models predicting distress, it only explained 2% more variance in distress than the model with loneliness (Table D3, Appendix D), Δ*R*^2^ = .024, *F*(2, 865) = 17.99, *p* < .001. This underscores the importance of loneliness as a predictor for distress.

### The Role of Prior Mental Health Conditions

The ANOVA revealed a small effect of prior mental health in loneliness, *F*(2, 883) = 20.00, *p* < .001, ω^2^ = .041, 95% CI [.02, .071]. Post hoc comparisons revealed that participants with current (*M* = 3.28, *SD* = 0.95) and past mental health conditions (*M* = 3.09, *SD* = 0.93) were significantly lonelier than those without (*M* = 2.77, *SD* = 0.88).

As for differences in emotion regulation by mental health status, no significant difference was found in suppression, *F*(2, 883) = 1.78, *p* = .169. There was also no significant difference in reappraisal, *F*(2, 883) = 0.59, *p* = .556.

As for the overall score regarding health behaviors, there was no significant difference between participants with past, current, or no mental health conditions, *F*(2, 883) = 0.57, *p* = .564. Investigating each behavior individually, we found a small difference in exercising regularly between participants depending on their mental health history, *F*(2, 883) = 6.73, *p* = .001, ω^2^ = .013, 95% CI [.002, .033]. Post hoc comparisons revealed that participants with no mental health conditions (*M* = 2.9, *SD* = 0.99) reported to exercise more regularly than participants with current (*M* = 2.65, *SD* = 1.05) or past (*M* = 2.6, *SD* = 1.03) mental health conditions. There was also a small difference in mindfulness practice by mental health group, *F*(2, 883) = 11.05, *p* < .001, ω^2^ = .022, 95% CI [.007, .047]. However, participants with no mental health conditions (*M* = 1.88, *SD* = 0.9) reported significantly less mindfulness practice than participants with current (*M* = 2.2, *SD* = 0.91) or past (*M* = 2.19, *SD* = 1) mental health conditions.

## Discussion

Our first hypothesis, anticipating lower psychological distress in 2023 compared to 2020, was not supported. Confirming the second hypothesis, loneliness and emotion regulation emerged as significant predictors for distress. The third hypothesis, positing differences by prior mental health, was only partially confirmed. Comparing levels of psychological distress in a three-year interval, we found that students reported higher distress in 2023 than in 2020, with 44.5% of students psychologically distressed, as compared to 27% in 2020. Certain health behaviors, i.e., keeping regular routines, getting sufficient sleep, and exercising regularly, were associated with lower distress. Identifying as male was associated with lower distress, experiencing past or current mental health conditions with higher distress. Beyond person-related variables and health behaviors, suppression and loneliness were predictive of higher psychological distress, while reappraisal predicted lower distress. Overall, loneliness was the most influential predictor for psychological distress and 41% of variance in distress was explained. Students identifying as non-binary reported the highest levels of loneliness, followed by female students. Students with prior or current mental health conditions reported more loneliness than others. There were no differences in emotion regulation by mental health history. Regarding health-related behaviors, students without prior mental health issues reported to exercise more regularly. However, students with previous and current mental health conditions reported practicing more mindfulness.

Our research design does not allow for causal attributions. However, our results are in line with existing research suggesting a deterioration of mental health in Germany during the later pandemic (Mauz et al., 2023; Walther et al., 2023). A US study highlighted an increase in severe levels of depression, anxiety, and stress among university students compared to previous years (Emmerton et al., 2024). However, the authors report this as part of a longer-term trend and identify academic performance as key stressor alongside several non-pandemic stressors (Emmerton et al., 2024). Our results might also capture differences in daily stressors, as the 2020 assessment occurred during lockdown and the semester break. Furthermore, global stressors beyond the pandemic must be considered (Mauz et al., 2023). Our exploratory analyses indicate that students in 2023 felt markedly burdened by stressors such as the climate crisis and the war in Ukraine. However, the reliance on self-developed self-report measures is a limitation.

Another limitation of this study lies in the use of a convenience sample. The study may have particularly attracted the interest of students struggling with mental health. However, a similar prevalence of mental disorders has been reported in other studies (e.g., Auerbach et al., 2018). Age distributions in both samples were comparable and in line with nationwide representative surveys (e.g., Statista, 2024). A notable 62% of participants identified as female (67% in 2020) and 3% as non-binary (1% in 2020). As the percentages of female and male students in Germany are approximately equal (Kroher et al., 2023) and since the pandemic had differential effects on male, female, and non-binary individuals (Flor et al., 2022), this limits the generalizability of our findings. Furthermore, demographic variables that may explain differences in mental health were not assessed due to anonymity considerations, thereby limiting comparability. Such variables could be financial status (e.g., Chandola et al., 2022), ethnic minority status (Plenty et al., 2021), and geographical location, as COVID-19 measures varied across regions in Germany. Finally, almost 10% of students reported being impacted by lasting consequences of a prior infection with COVID-19 (see Appendix B, Supplementary Materials).

### The Central Role of Loneliness in Predicting Psychological Distress

Loneliness was the strongest predictor for psychological distress. One explanation is to understand loneliness as a persisting consequence of the pandemic. Werner and colleagues (2021) reported loneliness being highly predictive of mental health issues in a longitudinal study, with loneliness during the pandemic only marginally predicted by pre-pandemic loneliness. Greater loneliness was reported in regions with more pandemic-related restrictions in a Norwegian study among students, however, the study also found that loneliness was linked to time spent on campus and declined again from 2021 to 2022 (Hysing et al., 2023). Conversely, loneliness could stem from existing mental health conditions. Students with diagnosed mental health conditions might struggle to seek social support or engage in fulfilling interactions (Perese & Wolf, 2005). As mental health conditions remain a sensitive topic, they may hesitate to confide in others (Schnyder et al., 2017; Schomerus et al., 2019).

Regarding gender differences in loneliness, our results align with existing findings (Hysing et al., 2023; Werner et al., 2021). A rise in female loneliness during COVID-19 has been linked to declining wellbeing (Lepinteur et al., 2022), likely due to gender gaps in other areas, e.g., finances or caretaking (Flor et al., 2022). However, the 3% non-binary students in our sample reported the highest loneliness. This percentage is in line with other German and international surveys (e.g., Ipsos, 2021). Transgender and non-binary individuals are more likely to experience discrimination and violence (Aparicio-García et al., 2018), with social support and LGBTQIA+ communities playing a vital role in mitigating negative mental health outcomes (Weinhardt et al., 2019). Thus, they may have been disproportionately affected by social restrictions. Alternatively, loneliness may have been high in this group before the pandemic (Aparicio-García et al., 2018).

Despite the importance of emotion regulation in psychopathology, we found no differences by mental health status. This may reflect the limitations of our narrow assessment, with adaptive emotion regulation increasingly seen as flexible (e.g., Aldao et al., 2015) and influenced by factors like strategy access and emotional awareness (Gratz & Roemer, 2004). In line, cognitive control and flexibility moderated the association between uncertainty intolerance and emotion regulation difficulties in a multi-wave pandemic study, affecting mental health (Godara et al., 2023). Our finding that health behaviors served as adaptive coping strategies generally aligned with previous research (Chen et al., 2020; Mata et al., 2021; Oftedal et al., 2019).

### Conclusion

Psychological distress was high in this university student sample even after the pandemic, with loneliness notably prevalent in the most distressed individuals. This underscores the importance of addressing loneliness in young adults. We also found that adaptive emotion regulation and specific health behaviors, such as adequate sleep, exercise, and maintaining daily routines, were associated with better mental health cross-sectionally. Future research should monitor loneliness among university students longitudinally. Specific interventions could address loneliness (Ma et al., 2020; Masi et al., 2011). Targeted programs in universities would benefit from particularly focusing on non-binary and female students. Counseling services could facilitate support groups and therapy referrals, and universities could expand practical assistance for challenges commonly faced by female students, such as caregiving responsibilities. By addressing mental health openly and fostering support systems, significant strides may be made towards reducing loneliness among university students.

## Supplementary Materials

The Supplementary Materials contain the following items:

- *Preregistration* (Hunsmann & Kühne, 2023S)
- *Research data and analysis code* (Hunsmann & Kühne, 2024S)
- *Online appendices* (Hunsmann et al., 2025S):
  - Appendix A: COVID-19 Pandemic Restrictions in Germany
  - Appendix B: Lasting Consequences of Prior COVID-19 Infections
  - Appendix C: Item List of Health Behaviors
  - Appendix D: Regression tables with detailed statistics for multiple regression models: a baseline model, a model with emotion regulation, a model with loneliness, and a model with all predictors

HunsmannJ. J.
KühneF.
 (2023S). University students’ mental health in the aftermath of the COVID-19 pandemic
[Preregistration]. PsychOpen. 10.17605/OSF.IO/97WTU


HunsmannJ. J.
KühneF.
 (2024S). University students’ mental health in the aftermath of the COVID-19 pandemic
[Research data and analysis code]. PsychOpen. 10.17605/OSF.IO/EH8U7


HunsmannJ. J.
WeckF.
WendtJ.
KühneF.
 (2025S). Supplementary materials to "Loneliness and distress in the aftermath of the COVID-19 pandemic: A cross-sectional study of German university students"
[Online appendices]. PsychOpen. 10.23668/psycharchives.16221
PMC1215222640510541

## Data Availability

The analysis code and data from this study are available on OSF (Hunsmann & Kühne, 2024S), the comparison data is available from the corresponding author upon request. No further materials were used.

## Preregistration

The study and analyses were conducted as previously registered (Hunsmann & Kühne, 2023S, March 31).

## Reporting Guidelines

JARS-Quant Reporting Standards for Studies Using No Experimental Manipulation
